# Xanthohumol Induces Growth Inhibition and Apoptosis in Ca Ski Human Cervical Cancer Cells

**DOI:** 10.1155/2015/921306

**Published:** 2015-04-09

**Authors:** Wai Kuan Yong, Sri Nurestri Abd Malek

**Affiliations:** Institute of Biological Sciences, Faculty of Science, University of Malaya, 50603 Kuala Lumpur, Malaysia

## Abstract

We investigate induction of apoptosis by xanthohumol on Ca Ski cervical cancer cell line. Xanthohumol is a prenylated chalcone naturally found in hop plants, previously reported to be an effective anticancer agent in various cancer cell lines. The present study showed that xanthohumol was effective to inhibit proliferation of Ca Ski cells based on IC_50_ values using sulforhodamine B (SRB) assay. Furthermore, cellular and nuclear morphological changes were observed in the cells using phase contrast microscopy and Hoechst/PI fluorescent staining. In addition, 48-hour long treatment with xanthohumol triggered externalization of phosphatidylserine, changes in mitochondrial membrane potential, and DNA fragmentation in the cells. Additionally, xanthohumol mediated S phase arrest in cell cycle analysis and increased activities of caspase-3, caspase-8, and caspase-9. On the other hand, Western blot analysis showed that the expression levels of cleaved PARP, p53, and AIF increased, while Bcl-2 and XIAP decreased in a dose-dependent manner. Taken together, these findings indicate that xanthohumol-induced cell death might involve intrinsic and extrinsic apoptotic pathways, as well as downregulation of XIAP, upregulation of p53 proteins, and S phase cell cycle arrest in Ca Ski cervical cancer cells. This work suggests that xanthohumol is a potent chemotherapeutic candidate for cervical cancer.

## 1. Introduction

Cervical cancer is a global health problem affecting women. According to the available data, 99.7% of all cervical carcinomas occur due to infection by human papillomavirus (HPV), especially HPV-16 and HPV-18, which World Health Organization identified as high risk carcinogenic agents. HPV affects body cells by integrating with the host's genome and inducing cellular dysregulation, such as increased DNA synthesis, cell proliferation, and cellular response to growth and differentiation factors, which eventually lead to the development of cervical cancer [1]. Two viral genes, E6 and E7, are expressed in HPV-positive cervical cancer cells. Their gene products are known to activate telomerase, prevent death of human primary epithelial cells, and inactivate major tumor suppressors (p53 and pRB proteins) [2]. Despite the growing availability of HPV vaccines, screening tests, and approved therapies, cervical cancer remains highly prevalent among women worldwide, ranking fourth, after breast, colorectal, and lung cancers [3].

A number of molecularly targeted agents were reported to modulate angiogenesis, growth factor receptors, cell cycle, and inflammation in cervical cancer signaling pathways. Amongst these are the chemotherapeutic agents currently used as advanced and metastatic cervical cancer treatment options, such as cisplatin, paclitaxel, topotecan, cetuximab, and bevacizumab. However, at present, the use of these agents results in medical complications and different grades of toxicities, such as nausea, vomiting, pain, fatigue, and anemia [4]. Thus, an effective and safe therapy for cervical cancer is urgently needed.

**Figure 1:**
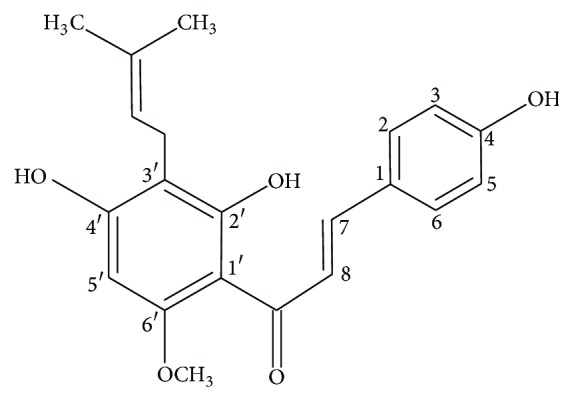
Xanthohumol structure.

Xanthohumol (Figure 1), a prenylated chalcone isolated from the female hop plant,* Humulus lupulus*, was reported to have* in vitro* antiproliferative and apoptosis-inducing properties on prostate, ovarian, breast, and endometrium cancer cell lines [5]. It has been posited that xanthohumol might provide therapeutic strategies against hormone-dependent breast cancer by suppressing breast cancer cell survival [6]. Inhibition of DNA synthesis, induction of cell cycle arrest in S phase, apoptosis, and cell differentiation were previously reported to be mediated by xanthohumol on MDA-MB-435 human mammary adenocarcinoma cells [7]. In addition, xanthohumol modulated the alkaline phosphatase isoenzymes that were expressed in malignant tissues [8] and was able to inhibit production of inflammatory factors, DNA synthesis and angiogenesis in MCF-7 cells, and breast cancer xenografts in mice [9]. Both death receptor and mitochondrial apoptosis pathways were also reported to be activated by xanthohumol. In addition, apoptosis induced by xanthohumol also involves endoplasmic reticulum stress and unfolded protein response. According to various extant reports, xanthohumol is involved in the regulation of antiapoptotic, proapoptotic proteins, and procaspases [5].

Vogel et al. [10] examined the cytotoxicity of xanthohumol on another cervical cancer cell line, HeLa, reporting an IC_50_ value of 9.4 ± 1.4 *μ*M following 72-hour long treatment. Szliszka et al. [11] reported that xanthohumol induced apoptotic cell death in HeLa cells via the expression of the tumor necrosis factor-related apoptosis-inducing ligand (TRAIL). In their study, xanthohumol, combined with TRAIL, was able to increase the percentage of apoptotic cells by increasing TRAIL-R2 protein levels on the cell surface. Although HeLa and Ca Ski are both cervical cancer cell lines, they differ in the content of papillomavirus strains. More specifically, HeLa contains human papillomavirus 18 (HPV-18), whereas Ca Ski contains both HPV-18 and HPV-16 [12], a higher copy number of HPV, as well as different genomic and proteomic profiles compared to HeLa [13]. Despite numerous studies in this field, apoptotic mechanism of xanthohumol on Ca Ski cervical cancer cell line has not yet been elucidated. In order to address this gap in the extant knowledge, in the present study, we aimed to elucidate the cytotoxic effects of xanthohumol on Ca Ski cervical cancer cells and the potential molecular mechanisms underlying its apoptotic-inducing activity.

## 2. Materials and Methods

### 2.1. Chemicals and Materials

Xanthohumol (purchased from BioBioPha Co., Ltd., China) was dissolved in dimethyl sulfoxide (DMSO) to prepare a stock solution of 10 mg/mL and 40 mg/mL. Both stock solutions were subsequently diluted to various concentrations with 10% fetal bovine serum- (FBS-) supplemented media, achieving the final concentration of 0.5% DMSO. Negative controls contained 0.5% DMSO in 10% FBS-supplemented media.

Ca Ski cells were obtained from the American Type Culture Collection (ATCC, USA), while acetic acid, doxorubicin hydrochloride, DMSO, sulforhodamine B (SRB), Hoechst 33342, propidium iodide (PI), Tris base, bromophenol blue, and bovine serum albumin (BSA) were purchased from Sigma Chemicals, USA. RPMI 1640 medium, fetal bovine serum (FBS), accutase, penicillin/streptomycin, and amphotericin B were obtained from PAA Laboratories, Austria. RNase was purchased from Life Technologies, USA. Trichloroacetic acid (TCA), ethanol, glycerol, hydrochloric acid (HCl), N-N′-methylenebisacrylamide, sodium dodecyl sulphate (SDS), *β*-mercaptoethanol, ammonium persulfate (APS), and TEMED were purchased from Merck Millipore, USA. FITC annexin V apoptosis detection kit and JC-1 mitochondrial membrane detection kit were supplied by BD Biosciences, USA. APO-BrdU TUNEL assay kit was purchased from Molecular Probes, USA, while caspase-3, caspase-8, and caspase-9 activity kits and anti-PARP-1 polyclonal primary antibody were purchased from GeneTex, USA. Phosphate buffered saline (PBS) and acrylamide were purchased from Nacalai Tesque, Japan, while Bradford reagent and BSA standard solutions were purchased from Bio-Rad laboratories, USA. All primary antibodies (except for PARP-1), secondary antibodies, RIPA lysis buffer, protease inhibitors, semi-dry transfer buffer, PBS-T wash buffer, and chemiluminescent substrate were obtained from Thermo Scientific, USA.

### 2.2. Cell Culture

Ca Ski cells were maintained in RPMI 1640 medium supplemented with 10% (v/v) heat-inactivated fetal bovine serum, 2% penicillin/streptomycin, and 1% amphotericin B. The cells were cultured in 5% CO_2_ incubator at 37°C in a humidified atmosphere.

### 2.3. Sulforhodamine B (SRB) Assay

This assay method is adapted from Houghton et al. [14]. In our study, 8,000 cells/well were seeded into a 96-well plate and incubated overnight for adherence. At the end of the incubation period, medium contained in each well was discarded and replaced by various concentrations of xanthohumol or doxorubicin and retained for 24-hour, 48-hour, and 72-hour treatment periods. At the end of these treatment periods, 50 *μ*L of 40% trichloroacetic acid (TCA) (w/v) was added to each well. The plates were incubated for 1 hour at 4°C before discarding the supernatant. Each well was subjected to five washes with 50 *μ*L of distilled water before being air-dried. Next, 50 *μ*L of SRB dye (0.4% w/v in 1% acetic acid) was added to each well and incubated for 30 minutes at room temperature for the staining process. To remove the unbound dye, each well was washed with 50 *μ*L of 1% acetic acid five times before being air-dried. Next, 100 *μ*L Tris-base (10 mM unbuffered, pH 10.5) was added to each well. The plate was shaken at 500 rpm for 5 minutes to solubilize the remaining bound SRB dyes. Absorbance values were measured at 492 nm using Synergy H1 Hybrid. This enabled the IC_50_ values to be determined by plotting dose response curves using percentage of inhibition of cell proliferation against treatment concentrations.

### 2.4. Apoptosis Detection by Annexin V-FITC/PI Staining

FITC annexin V apoptosis detection kit was used to detect apoptosis. Cells were seeded in six well plates (2.4 × 10^5^ cells/well) and allowed to adhere overnight. Next, the cells were treated with 10, 20, 30, and 40 *μ*M of xanthohumol for 24, 48, and 72 hours. The cells were harvested, washed, and resuspended in 1x binding buffer according to manufacturer's instruction. In the next step, 1 × 10^5^ cells were stained with 5 *μ*L of FITC-annexin V and 5 *μ*L of propidium iodide for 15 minutes. At the end of the staining process, 200 *μ*L of 1x binding buffer was added to each sample. Unstained and single-stained untreated cells were also included as controls. The number of 10,000 events was acquired for each replicate using Accuri C6 flow cytometer.

### 2.5. Morphological Studies

For phase contrast microscopy study, Ca Ski cells seeded in 24-well plates (5 × 10^4^ cells/well) were treated with 10, 20, 30, and 40 *μ*M xanthohumol for 48 hours. Upon discarding the media, the cells were washed twice with PBS before being observed under AxioCam ERc5s inverted phase contrast microscope.

Hoechst/PI fluorescent staining was carried out to observe changes in cell nucleus. 1.0 × 10^6^ cells were seeded in each 6 cm culture dish and allowed to adhere overnight. The cells were subsequently treated with various concentrations of xanthohumol for 48 hours. At the end of the incubation period, the media in the culture dishes were discarded and the cells were washed twice with PBS, after which 1 mL of PBS was added to each culture dish. To stain the cells with fluorescent dyes, 100 *μ*L of Hoechst solution (100 *μ*g/mL) and 25 *μ*L PI solution (100 *μ*g/mL) were added to each culture dish and incubated for 15 min. Finally, the cells were photographed using Leica DM-16000B fluorescent microscope.

### 2.6. Changes in Mitochondrial Membrane Potential Assay

JC-1, a lipophilic and cell-permeable fluorochrome, was used to measure mitochondrial membrane potential (Δ*ψ*_m_) according to the manufacturer's instructions. In preparation for this measurement, the cells seeded in 6-well plates were treated with 10, 20, 30, and 40 *μ*M of xanthohumol for 48 hours. At the end of the treatment, the cells were harvested and incubated with JC-1 working solution for 15 min. Next, the cells were washed and resuspended in 1x assay buffer. Finally, the intracellular fluorescence signal intensity of JC-1 was measured by Accuri C6 Flow Cytometer.

### 2.7. DNA Fragmentation by TUNEL Assay

DNA strand breaks in apoptotic cells were detected by APO-BrdU TUNEL assay kit. After incubation with xanthohumol at 10, 20, 30, and 40 *μ*M for 48 hours, cells were harvested and washed with PBS. Next, the cells were fixed with 1% (w/v) paraformaldehyde for 15 min, washed with PBS, and fixed with 70% (v/v) ethanol overnight. Ethanol was removed by centrifugation and DNA labeling steps were performed according to manufacturer's instructions. Samples were analyzed by flow cytometer and 10,000 events were acquired for each replicate.

### 2.8. Cell Cycle Analysis

After 48-hour treatment with xanthohumol, adherent and floating cells were collected, centrifuged, and fixed in 70% ethanol overnight. All samples were centrifuged to remove ethanol and the cell pellets were washed with cold PBS. The cells were subsequently resuspended in 300 *μ*L of staining solution containing final concentrations of 50 *μ*g/mL PI, 50 *μ*g/mL RNase, 0.1% Triton X-100, 1 mg/mL sodium citrate and distilled water. Following 30-minute long incubation at room temperature, cells were analyzed using flow cytometer and data analysis was performed by Modfit LT software.

### 2.9. Caspase-3, Caspase-8, and Caspase-9 Activity

Caspase activity assays were performed according to the manufacturer's instruction. Cells were seeded at a density of 1 × 10^6^ cells per culture dish. After being subjected to treatment for 48 hours, the cells were detached with accutase, washed, and resuspended in PBS. Next, 300 *μ*L of each sample was aliquoted into centrifuge tubes, after which 1 *μ*L of fluorescent substrate was added to each tube and incubated for 1 h at 37°C incubator. At the end of the incubation period, the cells were centrifuged at 3000 rpm for 5 minutes and the supernatant was removed. In the next step, the cells were resuspended in 0.5 mL wash buffer and centrifuged at 3000 rpm for 5 minutes. This step was repeated before performing the analysis with fluorescence plate reader. In preparation for this step, the cell pellets were resuspended in 100 *μ*L wash buffer and the cell suspension was transferred to each well of the black microtiter plate. Fluorescence intensity was measured at excitation wavelength = 485 nm and emission wavelength = 535 nm. Unlabeled cells were used as blanks.

### 2.10. Western Blot Analysis

In preparation for this analysis, cells were seeded at a density of 1 × 10^6^ cells per culture dish. After 48-hour long treatment, whole cell lysate was prepared by washing the cells with cold PBS and lysed with cold RIPA buffer containing protease inhibitors. Protein concentrations were measured using Bradford assay with bovine serum albumin as the standard. To denature the protein samples, lysed samples were mixed with 4x Laemmli buffer (at 3 : 1 ratio) and boiled at 100°C for 7 minutes. The 4x Laemmli buffer consisted of final concentrations of 0.25 M Tris-HCl pH 6.8, 8% SDS, 10% *β*-mercaptoethanol, 30% glycerol, and 0.03% bromophenol blue. Next, 40 *μ*g protein per lane was separated by sodium dodecyl sulfate polyacrylamide gel electrophoresis (SDS-PAGE) using 14% resolving gel and 4% stacking gel. Proteins were transferred to 0.2 *μ*m nitrocellulose membrane using semi-dry method (25 V for 9 minutes) followed by membrane blocking with 1% BSA in PBS-T using SNAP i.d. protein detection system. Primary and secondary antibodies were incubated for 15 and 10 minutes, respectively, with gentle agitation at room temperature. Cold blocking buffer (1% BSA in PBS-T) was used to dilute both the primary and secondary antibodies. After being probed by primary and secondary antibodies, the protein membrane was incubated with chemiluminescent substrates for 5 minutes. Finally, protein bands on the membranes were visualized under Vilber Fusion Fx-7 CCD camera.

### 2.11. Statistical Analysis

All values reported here are shown as mean ± standard error of the mean (SEM) and all experiments were performed at least twice using sample triplicates. Figures from morphological studies, flow cytometry plots, and Western blot analyses are representative of the experiment replicates. Comparisons between control and treated groups were conducted using one-way ANOVA with post hoc Tukey test (*P* < 0.05 was considered statistically significant). All calculations were performed using Microsoft Excel and SPSS version 17.0.

## 3. Results

### 3.1. Xanthohumol Decreased Proliferation and Induced Apoptosis on Ca Ski Cells

**Figure 2:**
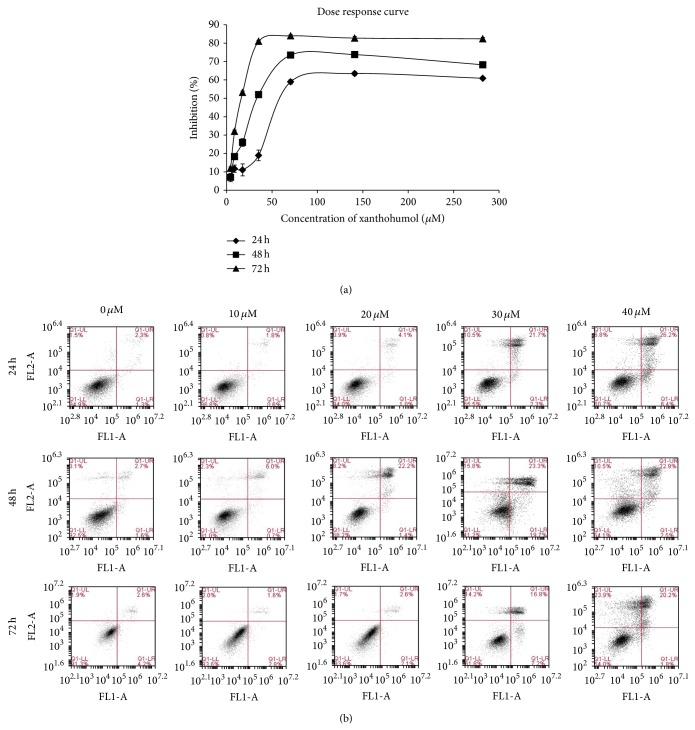
Xanthohumol reduced Ca Ski cell proliferation and induced apoptosis. (a) Dose response curve of the percentage of inhibition on the cells against different concentrations of xanthohumol (0–282 *μ*M, equivalent to 0–100 *μ*g/mL) following 24-hour, 48-hour, and 72-hour treatment. (b) The effects of xanthohumol on apoptosis by annexin V-FITC/PI staining. The 1st quadrant (lower left-LL) depicts percentage of viable cells, the 2nd quadrant (lower right-LR) depicts % of early apoptotic cells, the 3rd quadrant (upper right-UR) depicts late apoptotic or secondary necrotic cells, and the 4th quadrant (upper left-UL) depicts primary necrotic cells. Xanthohumol increased the percentage of late apoptotic or secondary necrotic cells (in UR quadrant).

Xanthohumol induced cell death and reduced cell proliferation of Ca Ski cells in dose- and time-dependent manner. Dose response curves shown in Figure 2(a) indicate that the growth inhibition increased with treatment dose and time. Notably, xanthohumol induced highest inhibition and lowest IC_50_ value when the treatment period was extended to 72 hours. More specifically, treatments lasting 24, 48, and 72 hours yielded IC_50_ values (i.e., the concentration resulting in 50% growth inhibition) of 59.96 ± 1.95, 34.01 ± 1.13, and 20.08 ± 1.12 *μ*M, respectively. In comparison, doxorubicin used as a reference compound resulted in IC_50_ values of 14.16 ± 0.34 *μ*M, 0.86 ± 0.13 *μ*M, and 0.18 ± 0.00 *μ*M, following 24, 48, and 72 hours of treatment, respectively.

Figure 2(b) shows the annexin V/PI density plot of xanthohumol on Ca Ski cells. As can be seen, xanthohumol did not induce significant increase in early apoptotic cells in Ca Ski cells. Percentage of early apoptotic cells (Q1-LR quadrant) was highest when the cells were treated with 30 *μ*M xanthohumol (19.7%) for 48 hours. However, other doses and treatment times resulted in 0.6–7.2% of early apoptotic cells appearing at Q1-LR quadrant. On the other hand, the percentage of late apoptotic/secondary necrotic cells (Q1-UR quadrant) increased significantly after only 24 hours of treatment—from 2.3% in untreated cells to 26.2% in cells treated with 40 *μ*M xanthohumol. After 48 hours of treatment with 20, 30, and 40 *μ*M xanthohumol, the percentage of late apoptosis/secondary necrosis cells in the population exceeded 20%.

Upon completion of 72-hour treatment with xanthohumol, most Ca Ski cells subjected to high concentrations (20, 30, and 40 *μ*M) detached from the culture plate, shrunk in size, and underwent extensive vacuolation and cell death. Many cells appeared as debris in the first density plot (FSC versus SSC plot) collected in flow cytometry. Debris was excluded from the quadrant analysis in FL1 versus FL2 plots. In addition, in order to avoid collecting large number of dead cells, the samples subjected to 72-hour treatment were excluded from the subsequent analyses. This decision was made as TUNEL assay and cell cycle study require high number of live cells for analysis. The 48-hour treatment resulted in lower percentage of dead or secondary necrotic cells (in Q1-UL quadrant) compared to that obtained after 72 hours of treatment. Hence, the samples subjected to 48 hours of treatment with xanthohumol were selected for the subsequent analyses.

### 3.2. Xanthohumol Induced Morphological Changes and Loss of Mitochondrial Membrane Potential (Δ*ψ*_m_)

**Figure 3:**
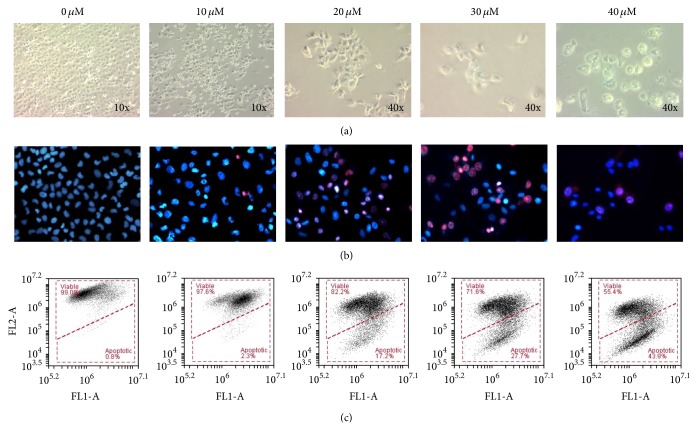
(a) Decreased number of cells, changes in cell shape, and vacuolation were observed in cells treated with xanthohumol (at 10x and 40x magnification). (b) After staining with Hoechst and PI, cells treated with xanthohumol showed an increase in the number of brightly stained nuclei and nuclear membrane permeability (40x magnification). In untreated cells, a greater number of nuclei were observed. The nuclei stained red by PI indicated dead cells with permeabilized membrane. The treatment with 40 *μ*M xanthohumol resulted in greatly reduced number of cells. (c) Changes in mitochondrial membrane potential of Ca Ski cells. Xanthohumol treatment resulted in a shift in mitochondrial membrane potential from higher to lower FL2-A signals, where JC-1 remained as monomers and emitted intense green fluorescence.

As shown in Figure 3(a), untreated Ca Ski cells are characterized by high confluency, close arrangement between cells, and polygonal shape. Treatment with xanthohumol resulted in a reduction in cell number, detachment of cells, atrophy, and size shrinkage.

Fluorescence microscopy using Hoechst 33342 and propidium iodide (PI) dyes revealed the nuclear morphology of Ca Ski cells (Figure 3(b)). In the negative control group, cell nuclei were dim and dark blue in color. In contrast, cells treated with 10 *μ*M xanthohumol had brightly stained, condensed, and fragmented nuclei. Most of the nuclear membranes were still intact, as only a small number of nuclei were stained red. At higher xanthohumol concentrations, a greater number of nuclei assumed red color, indicating that the nuclear membranes were damaged, permeabilized, and stained by PI.

Change in mitochondrial membrane potential was also observed in Ca Ski cells treated with xanthohumol, especially at 20, 30, and 40 *μ*M (Figure 3(c)). Viable cells possess high Δ*ψ*_m_, and JC-1 monomers combine as aggregates that emit intense red fluorescence signal, as detected in FL2-A channel. In contrast, apoptotic cells possess reduced or depolarized Δ*ψ*_m_, where JC-1 monomers emit intense green fluorescence detected in FL1-A channel. After 48 hours of treatment, percentage of viable cells decreased from 99.0% to 55.4% for cells treated with 40 *μ*M xanthohumol, while the percentage of apoptotic cells increased from 0.8% to 43.9%.

### 3.3. Xanthohumol Induced DNA Fragmentation and Cell Cycle Arrest at S Phase

**Figure 4:**
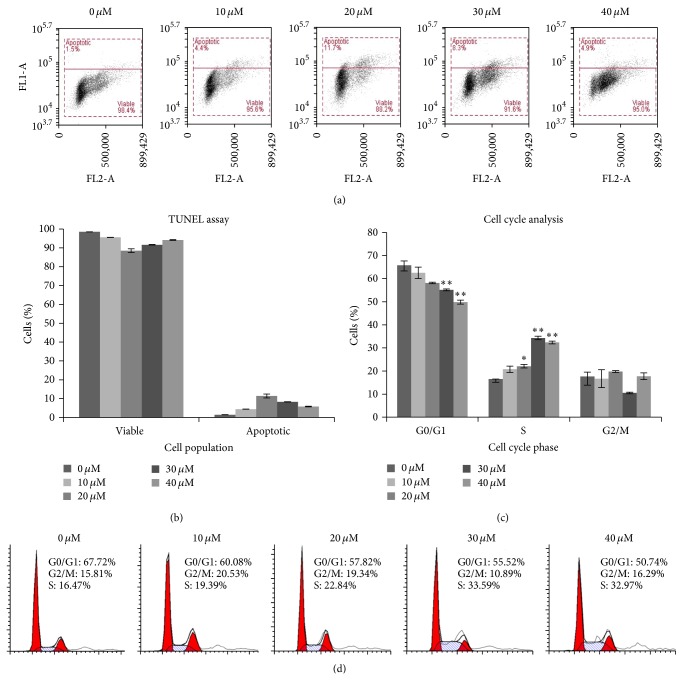
(a, b) TUNEL assay was used to detect DNA fragmentation. A small increase in the percentage of cells with fragmented DNA was observed especially following treatment with 20 *μ*M xanthohumol. (c, d) Cell cycle analysis of Ca Ski cells treated with xanthohumol. Percentage of cells arrested at S phase increased significantly, as revealed by ModFit software analysis. In the bar chart, statistical significance of the differences between the treatment and control results is marked with (^∗^) (*P* < 0.05) and (^∗∗^) (*P* < 0.01).

TUNEL (terminal deoxynucleotidyl transferase-mediated dUTP nick end labeling) assay was used to detect DNA fragmentation and identify individual cells that were undergoing apoptosis. In this study, no significant DNA fragmentation was observed in Ca Ski cells, although cells treated with 20 *μ*M had higher percentage of fragmented DNA (11.39 ± 1.02%) compared to untreated cells (1.44 ± 0.05%) and cells treated with 10 *μ*M (4.39 ± 0.07%), 30 *μ*M (8.25 ± 0.20%), and 40 *μ*M xanthohumol (5.77 ± 0.25%) (Figures 4(a) and 4(b)).

As can be seen in Figures 4(c) and 4(d), in Ca Ski cells, xanthohumol significantly induced cell cycle arrest at the S phase. More specifically, the percentage of cells at the S phase increased from 16.51 ± 0.04% for untreated cells to 20.78 ± 1.39%, 22.09 ± 0.75%, 34.33 ± 0.74%, and 32.33 ± 0.60% for cells treated with 10, 20, 30, and 40 *μ*M xanthohumol, respectively.

### 3.4. Xanthohumol Mediated Activation of Caspase-3, Caspase-8, and Caspase-9 and Changes in Protein Levels

**Figure 5:**
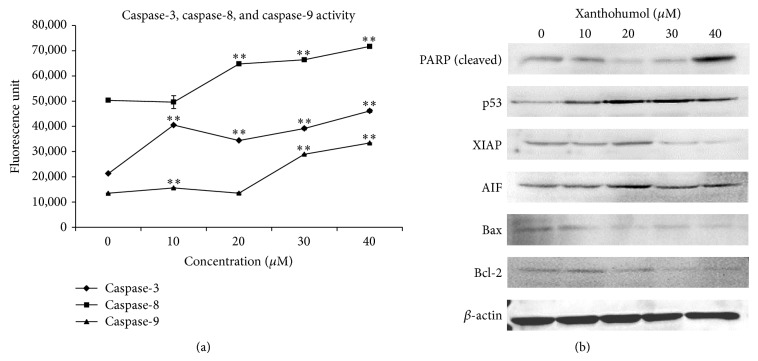
(a) Xanthohumol increased the caspase-3, caspase-8, and caspase-9 activity following 48 hours of treatment. Statistical significance of the differences between the treatment and control results is marked with (^∗^) (*P* < 0.05) and (^∗∗^) (*P* < 0.01). (b) Xanthohumol affects apoptosis-associated protein levels in Ca Ski cells. The anti-*β*-actin was used as a loading control.

To determine whether xanthohumol-induced apoptosis in Ca Ski cells is caspase-dependent, the activity of caspase-3, caspase-8, and caspase-9 was examined.  Figure 5(a) shows that xanthohumol increased the activity of caspase-3, caspase-8, and caspase-9 in Ca Ski cells after 48 hours of treatment. This finding indicates that apoptosis is caspase-3 dependent and might involve both the intrinsic and extrinsic pathways.

Changes in the expression of proteins involved in regulation of survival and cell death following xanthohumol treatment were examined by Western blot analysis. As illustrated in Figure 5(b), the level of cleaved PARP increased remarkably after the treatment with 40 *μ*M xanthohumol, while decreasing at lower concentrations (20 and 30 *μ*M). During apoptosis, PARP, a 116 kDa nuclear protein, is cleaved by caspase-3 or caspase-7 to yield 85 kDa and 25 kDa fragment. The primary antibody for PARP used in this study detects the N-terminal DNA-binding domain (25 kDa) of PARP. The level of p53, a tumor suppressor protein, increased notably in a dose-dependent manner. XIAP, AIF, Bax, and Bcl-2 are apoptosis-related proteins. The level of XIAP, a member of the inhibitors of apoptosis proteins (IAP) family, decreased following the treatment with 30 and 40 *μ*M xanthohumol. AIF, Bax, and Bcl-2 proteins are related to the apoptotic intrinsic pathway. The expression level of AIF, a prosurvival protein, slightly increased following the treatment with 20 *μ*M xanthohumol. Bcl-2, antiapoptotic protein, slightly decreased in Ca Ski cells treated with increasing concentrations of xanthohumol. Interestingly, the expression of Bax, a proapoptotic protein, decreased.

## 4. Discussion

To the best of the authors' knowledge, this is the first report of the cytotoxicity of xanthohumol on Ca Ski cervical cancer cell line. Our results show that xanthohumol induced cell death and morphological changes in Ca Ski cells, as revealed by phase contrast microscopy and fluorescent staining. In this study, changes in mitochondrial membrane potential were also observed. We noted a lower percentage of early apoptotic cells compared to late apoptotic and secondary necrotic cells. Moreover, a small number of cells with DNA fragmentation were observed. The cell cycle analysis revealed absence of the sub-G1 peak corresponding to fragmented DNA in apoptosis. This finding raised the question of the pathway induced by xanthohumol on Ca Ski cells, prompting further investigation.

Xanthohumol was effective in triggering S phase arrest in the cell cycle. S phase is defined as the specific period during the cell cycle when DNA synthesis is taking place, resulting in a double quantity of DNA per cell. The result obtained in this phase of our study is consistent with that reported by Lee et al. [15], whereby xanthohumol was found to induce cytotoxicity against A549 lung cancer cells via the inhibition of DNA topoisomerase I (Top1) activity. Top1 inhibitors are associated with S phase arrest in cell cycle by initiating DNA damage and causing replication fork arrest during DNA synthesis at the S phase [16].

Our data also indicated activation of caspase-3, caspase-8, and caspase-9, suggesting that this compound might induce both extrinsic and intrinsic pathways of apoptosis. Activation of both death receptor- and mitochondrial-mediated pathways by xanthohumol has been reported for human colon cancer, thus supporting our findings [17]. Xanthohumol was also shown to mediate changes in the levels of cleaved PARP, p53, XIAP, AIF, and Bcl-2 proteins. PARP is a nuclear protein involved in DNA repair mechanism. As observed in Figure 5(b), PARP expression increased after the treatment with 40 *μ*M xanthohumol and was relatively lower at 20 and 30 *μ*M concentrations. This observation is comparable to the DNA fragmentation data shown in Figure 4(a), in which treatment with 20 and 30 *μ*M xanthohumol produced 11.7% and 8.3% of fragmented cells, respectively. Thus, we hypothesize that, at these concentrations, PARP was actively expressed to counter DNA fragmentation, hence lowering the expression of cleaved PARP in cells treated with 20 and 30 *μ*M xanthohumol. Cleavage of PARP is executed by caspases and is recognized as an indicator of caspase-dependent apoptosis. Caspase-3 activity was the highest at 40 *μ*M, which might lead to increased cleavage of PARP. Xanthohumol was reported to induce PARP cleavage in human breast cancer cell lines (MCF-7 and T47-D)* in vitro* [18].

Xanthohumol is known to induce upregulation of p53 in cancer cells* in vitro* [5]. It is widely accepted that p53 tumor suppressor proteins are targets of high risk HPV E6 oncoprotein, which binds and inactivates p53, resulting in deregulation of cell cycle and mutation [19]. Thus, upregulation of p53 by xanthohumol might reflect the inhibition of this relationship. XIAP, a member of inhibitor of apoptotic proteins involved in cell death regulation and cell cycle, halts activation of caspase-3, caspase-7 and caspase-9 by direct inhibition. Inactivation of XIAP prevents inhibition and protects stability of executioner caspases, subsequently promoting the onset of apoptosis [20]. Apoptosis induced by xanthohumol, which is correlated with the downregulation of XIAP, was also reported in leukemia cells* in vitro* [21]. IAP target strategies are widely studied in anticancer therapies.

AIF cleavage is regulated by intracellular ROS level and activation of calpains, a family of cysteine proteases. Cleaved AIF proteins are released from the intermembrane space of mitochondria into the cytosol through Bax/Bcl-2 pores to play vital role in DNA fragmentation and chromatin condensation [22]. Xanthohumol was reported to target mitochondrial-mediated apoptosis by increasing ROS production, disruption of cellular redox balance, and mitochondrial integrity [23]. In this study, the expression level of AIF increased slightly following the treatment with 20 *μ*M xanthohumol.

During mitochondria-mediated apoptosis, disruption of mitochondrion may lead to the onset of the following cascade: activation of caspase-9, loss in mitochondrial membrane potential, and cleavage of Bcl-2 protein [24]. In this study, caspase-9 activity significantly increased in Ca Ski cells treated with 30 and 40 *μ*M xanthohumol. The same xanthohumol concentrations (30 and 40 *μ*M) resulted in loss of mitochondrial membrane potential and decreased level of Bcl-2 protein in treated cells. Based on these findings, it can be concluded that xanthohumol-induced cell death might involve the intrinsic apoptosis mechanism. Interestingly, Bax, a proapoptotic protein that is commonly upregulated in apoptosis, was observed to be downregulated. This finding is in-line with the results of several extant studies showing that Bax-independent mitochondria-mediated death can be induced by several natural products. Resveratrol—a polyphenol present in grapes, wine, and peanuts—was documented to induce apoptosis in Bax-mediated and Bax-independent intrinsic apoptosis pathway [25]. Gossypol, a polyphenolic compound isolated from cottonseed, and chelerythrine, found in the plant* Chelidonium majus*, are some examples of potential anticancer agents that induce apoptosis via Bax-/Bak-independent mitochondrial mechanism [26, 27]. Thus, we posit that xanthohumol-induced apoptosis may be Bax-independent.

Various potential therapeutic targets have been developed for cervical cancer, including anti-EGFR, hepatocyte growth factor- (HGF/C-) met inhibitors, insulin-like growth factor (IGF/IR) targeting agents, intracellular signaling kinases, cytotoxics, PARP inhibitors, epigenetics, estrogen receptor blockers, and biologic agents [28]. Xanthohumol was reported to modulate activity of estrogen synthase and decrease estrogen synthesis* in vitro* in breast cancer cells [6]. Extrinsic apoptosis was also reported in HeLa cervical cancer cells by enhancing TRAIL-induced apoptosis and increasing the expression of TRAIL-R2 in HeLa cells [11]. In addition to apoptosis-inducing properties, extant studies have demonstrated potential antiviral activity of xanthohumol, which is exhibited by inhibiting the replication of bovine viral diarrhea virus (BVDV), a surrogate model of hepatitis C virus (HCV) [29].

In previous studies, oral administration of xanthohumol to mice revealed no adverse effects on major organ and biochemical functions, such as carbohydrate, lipid, and protein metabolism [30]. Female reproduction and development of offspring remained normal upon administration of xanthohumol in rats [31]. A dietary study on menopausal women was conducted to evaluate safety and pharmacokinetics of xanthohumol through analysing the subjects' serum and urine samples after consuming hop extract capsules containing xanthohumol. No adverse effect was observed on sex hormones or blood clotting [32]. Overall, xanthohumol treatment shows good tolerance and selective toxicity* in vivo*, in addition to its promising anticancer properties in various cancer cell lines. Further investigations are nonetheless needed, with the aim of elucidating the detailed mechanism induced by xanthohumol in cervical cancer cell lines and HPV viruses. In conclusion, we propose that xanthohumol induced caspase-dependent cell death, apoptosis by both intrinsic and extrinsic pathways, and PARP cleavage, as well as upregulation of p53 tumor suppressor proteins and downregulation of XIAP in Ca Ski cervical epidermoid carcinoma cells.

## Abbreviations

*μ*M: Micromolar
AIF: Apoptosis-inducing factor
Bcl-2: B-cell lymphoma 2
Ca Ski: Cervical epidermoid carcinoma cells
h: Hour
p53: Tumor suppressor protein p53
PARP: Poly adenosine diphosphate- (ADP-) ribose polymerase
PI: Propidium iodide
SRB: Sulforhodamine B
XIAP: X-chromosome linked inhibitor of apoptosis protein.
